# Drug Reaction with Eosinophilia and Systemic Symptoms Syndrome in a Child with Cystic Fibrosis

**DOI:** 10.1155/2023/1006376

**Published:** 2023-02-02

**Authors:** Ahmed Abushahin, Haneen Toma, Sara G. Hamad, Mutasim Abu-Hasan

**Affiliations:** ^1^Department of Pediatrics, Pulmonology Division, Sidra Medicine, P.O. Box 26999, Doha, Qatar; ^2^Department of Pediatrics, Pulmonology Division, Hamad Medical Corporation, P.O. Box 3050, Doha, Qatar

## Abstract

**Background:**

Drug reaction with eosinophilia and systemic symptoms (DRESSs) syndrome is an idiosyncratic drug-induced reaction that rarely occurs in children but can lead to serious complications. It manifests most commonly with fever, extensive skin eruptions, and eosinophilia. Symptoms typically develop two to six weeks after the initiation of the inciting drug. Visceral organ involvement especially the liver can also occur and if not recognized early and the inciting drug is not stopped immediately, it can lead to liver failure. Therefore, early diagnosis is important but can be very challenging because of disease rarity, lack of a diagnostic test, and its overlap with other common pediatric allergic and infectious conditions. *Case Presentation*. A 2.5-year-old boy with known diagnosis of cystic fibrosis, bilateral bronchiectasis, pancreatic insufficiency, and chronic airway colonization with *Pseudomonas aeruginosa* was admitted to our hospital with acute pulmonary exacerbation of CF lung disease. He was treated with intravenous piperacillin-tazobactam and intravenous amikacin in addition to airway clearance. On day 18 of treatment, the patient developed high grade fever followed by diffuse erythematous and pruritic maculopapular rash. Blood tests showed high eosinophilia, high C-reactive protein (CRP), and high liver enzymes levels. The clinical features and the laboratory findings were consistent with the DRESS syndrome. Therefore, all antibiotics were discontinued. Progressive resolution of the symptoms was observed within two days. Laboratory abnormalities were also normalized in the follow-up clinic visit 4 months later.

**Conclusion:**

Our case demonstrates the importance of early recognition of the DRESS syndrome in children who develop fever and skin rashes with eosinophilia while undergoing long-term antibiotic treatment. Prompt discontinuation of the offending drug is the cornerstone therapy and results in the resolution of symptoms and prevention of serious complications.

## 1. Background

Drug reaction with eosinophilia and systemic symptoms (DRESSs) is a very rare but potentially severe drug-induced hypersensitivity reaction that can occur in children and adults [1]. The pathophysiology of DRESS is not completely characterized, but it is hypothesized to be multifactorial and results from a delayed T-cell-dependent allergic reaction to an inciting drug [2].

Patients with the DRESS syndrome usually present with fever, skin eruptions, and eosinophilia within days to weeks of drug exposure. The liver, the kidney, and the lung injury can also occur [3]. DRESS may rarely affect the heart but is associated with high mortality [4]. The degree of symptoms and the extent of organ involvement in patients with the DRESS syndrome can range from mild to severe. Substantial mortality can result from a severe disease and estimated at approximately 5% of all affected children and 10% of all affected adults [1, 5]. Death in patients with severe DRESS syndrome occurs mainly due to liver failure. Therefore, early recognition of the condition and immediate discontinuation of the inciting drug is paramount.

The diagnosis of DRESS syndrome can be easily overlooked, especially in children, because of its rarity and because of its overlap with other more common pediatric allergic, autoimmune, and infectious conditions [1, 6]. Therefore, clinicians should be aware of this condition in order to effectively treat the disease and prevent the development of serious complications.

We present here a case of a child with cystic fibrosis who was hospitalized with CF-related pulmonary exacerbations and developed acute symptoms and laboratory abnormalities characteristic of DRESS syndrome 18 days after the initiation of intravenous piperacillin-tazobactam and amikacin.

## 2. Case Presentation

The patient is a 2.5-year-old boy with confirmed diagnosis of cystic fibrosis and multiple CF-related comorbidities including pancreatic insufficiency, bilateral bronchiectasis, and chronic airway colonization with *Pseudomonas aeruginosa*. He was admitted to our hospital for acute pulmonary exacerbation of CF lung disease after presenting with fever and cough for 2 days. The patient was started on intravenous piperacillin-tazobactam (100 mg/kg/day divided three times daily) and amikacin (30 mg/kg/day once daily) based on the results of previously obtained induced-sputum culture. There is no previous history of any drug reaction to treatment with same antibiotics. The home therapy of airway clearance, oral pancreatic-enzyme replacement therapy, and nutritional supplements was continued. The patient's respiratory symptoms gradually improved with treatment. However, on day 18 of intravenous antibiotic treatment, the patient developed a high-grade fever (up to 39°C), followed by diffuse erythematous maculopapular pruritic rash a day later. The rash initially involved his face and his trunk but rapidly spread to his entire body. Facial edema was also noted. The rest of the physical examination was unremarkable. No lymph node enlargement, mucus membrane involvement, or organomegaly were noted.

Complete blood count (CBC) showed low white cell count (4.1 × 10^9^/L), low absolute neutrophil count (0.9 × 10^9^/L), and low platelet count (92 × 10^9^/L). However, differential WBC showed a significantly elevated eosinophil count with an absolute count of 1.8 × 10^9^/L. C-reactive protein (CRP) levels were also high (117 mg/dL).

Liver enzymes levels were elevated with increased aspartate aminotransferase (AST) and alanine aminotransferase (ALT) levels up to 178 IU/L and 686 IU/L, respectively (>12 times the upper normal limit). Renal function test including BUN and creatinine, as well as electrolyte levels, were all normal. Blood culture results were negative. Serum polymerase chain reaction (PCR) was positive for both Epstein–Barr virus (EBV) and cytomegalovirus (CMV) but negative for human herpes virus-6 (HHV-6), chlamydia, and *Mycoplasma pneumoniae*. Respiratory viral-PCR panel was negative for adenovirus, human metapneumovirus, influenza A-B, MERS-coronavirus, and parainfluenza-1–.

The DRESS syndrome was suspected based on the patient's symptoms and laboratory findings in the context of prolonged administration of intravenous antibiotics. The diagnosis was also supported by the Registry of Severe Cutaneous Adverse Reactions (RegiSCAR) scoring system. Our patient had a total score of 5 (Table1). Therefore, antibiotics were immediately discontinued. We also elected symptomatic treatment with diphenhydramine and close monitoring only, since there was no evidence of pulmonary, cardiac, or renal involvement, and since the hepatic involvement was mild. The patient showed gradual resolution of the fever and the skin rash within two days of antibiotic removal. At the same time, laboratory abnormities were also significantly improved with CRP of 35 mg/dL, ALT of 94 IU/L, and AST of 90 IU/L.

The drastic clinical and laboratory improvements after stopping antibiotics further supported the diagnosis of DRESS syndrome over other overlapping diseases in children from different infectious, hematological, or autoimmune etiologies. The skin patch test and the lymphocyte proliferation test were not performed.

**Figure 1:**
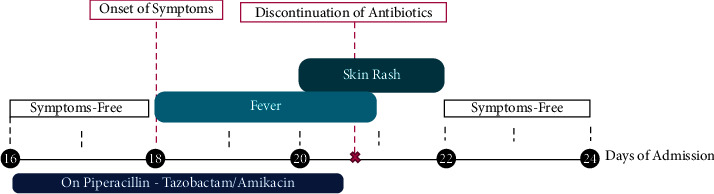
Patient timeline. The timeline displays the patient's course beginning from the initiation of the intravenous medications, subsequent symptoms, and the dramatic clinical resolution of symptoms after removal of the offending drugs.

The patient was eventually discharged on day 24 of admission in good condition and without any complications. The patient remained asymptomatic during the follow-up visits two months after discharge. All laboratory tests were completely normal on the follow-up visit at 4 months. Trends in the relevant laboratory findings are summarized in Table 2, and the timeline displaying the patient's course is shown in Figure 1.

## 3. Discussion and Conclusions

We presented a rare case of a child with DRESS syndrome, which is a potentially life-threateningdrug-induced hypersensitivity reaction with multisystem involvement. The diagnosis was made very early and resulted in resolution of symptoms and lab tests soon after stopping inciting drugs [1].

DRESS syndrome is very rare, with a reported prevalence of 2.8 : 100,000 in adults [7]. In the pediatric population, only isolated cases and few retrospective studies have been reported [8–10]. Therefore, its actual incidence in children has not yet been established. In general, DRESS syndrome occurs less frequently in children than adults and has a better prognosis, with a significantly lower mortality in children (5%) than in adults (10%) [1, 6].

DRESS syndrome is believed to result from drug‐specific T cell activation of eosinophils that appears within days to weeks of exposure to the culprit drug and leads to multisystemic manifestation [1, 2].

Approximately, 50 culprit drugs have been implicated in the development of DRESS syndrome, with anticonvulsants and antibiotics being the most common inciting agents [1, 3]. Kim et al., in a recent systematic review, identified the culprit medications in most pediatric cases as that in adult cases. Antiepileptic medications were the most implicated drugs (in 52.6% of cases), including carbamazepine, phenytoin, and lamotrigine, followed by antibiotics (33%) such as amoxicillin/clavulanate, vancomycin, and dapsone [6]. In our case, piperacillin-tazobactam and amikacin were the potential inciting drugs for DRESS syndrome. Moris et al. reported piperacillin-tazobactam in only two of the 103 cases of DRESS syndrome (2%) but was rarely reported by others [1, 5, 11]. To the best of our knowledge, amikacin has not been reported as a triggering drug for DRESS syndrome. Accordingly, DRESS syndrome in our case was very likely triggered by piperacillin-tazobactam. Causality can be supported by demonstrating a positive reaction to piperacillin-tazobactam using skin patch tests and/or in vitro tests (e.g., the lymphocyte proliferation test), both of which were not performed in our case.

The exact pathogenesis of DRESS syndrome is not well-characterized. A complex interaction between genetic susceptibility, aberrant drug-detoxification pathways, and the immune system, leading to a vigorous T cell-mediated hypersensitivity response to a specific drug, has been hypothesized [1, 2]. Studies on delayed drug reactions suggest that drug-specific CD4+ and CD8+ T-cell activation can preferentially stimulate and recruit eosinophils by releasing certain cytokines, such as IL5 [12]. Furthermore, the reactivation of several herpes viruses (HHV-6, HHV-7), EBV, and CMV, which coincide with the clinical symptoms of DRESS syndrome, has also been linked to its pathogenesis [12–16]. EBV- and CMV-PCR were positive in our case, indicating possible viral activation.

DRESS syndrome has a broad spectrum of clinical features that usually appear two to six weeks after exposure to an offending drug [5, 17]. Kim et al. demonstrated that the average time from drug exposure to the onset of symptoms in children with DRESS syndrome was 23.2 days (range: 0.42–112 days) [6]. The average age of diagnosis was 8.7 years old [6, 17].

DRESS syndrome displays a distinct phenotype characterized by a fever ≥38.5°C in 96%–100% of the cases, usually preceded by a skin eruption [6, 17]. Cutaneous eruptions present in 85%–100% of cases as diffuse, pruritic, or nonpruritic maculopapular/morbilliform. Other eruptions may be described as targetoid, urticarial, pustular, blistering, lichenoid, exfoliative, or eczematous lesions [1, 5, 17].

Lymphadenopathies are frequently described in 80% of the cases with DRESS syndrome [1]. Among the visceral organs, the liver is the most commonly involved (50%–84% of cases). The liver involvement ranges from the transient elevation of enzymes to fulminant hepatic failure, which is the primary cause of death in DRESS syndrome [5, 6]. Kidney injury (11%–57% of cases) can also range from proteinuria to renal failure [6, 17]. The lung involvement (2.6%–5% of cases) ranges from interstitial pneumonitis to acute respiratory distress syndrome [6, 18]. The cardiac involvement (i.e., myocarditis) has been rarely reported in cases of DRESS, and it was associated with 45.2% mortality rate [4]. Radovanovic et al. reported that abnormal electrocardiography was found in 71.4% of the patients, and depressed left ventricular ejection fraction was found in 45% of the patients with cardiac involvement [4].

Other systems involving the gastrointestinal tract (i.e., colitis or pancreatitis) and the central nervous system (i.e., encephalitis) have been less frequently reported [5, 6, 17]. Hematological abnormalities are commonly associated with this condition, including leukocytosis with peripheral eosinophilia, atypical lymphocytosis, and thrombocytopenia. Eosinophilia is typically the predominant abnormality and is found in 82%–95% of the cases [1, 5, 6]. These different clinical manifestations have been attributed to the specific chemical properties of each drug or its reactive metabolites.

Owing to the heterogeneity of clinical presentation, other disease mimickers should be excluded. These mimickers include, but are not limited to, toxic shock syndrome, Stevens– viral infections, infectious mononucleosis particularly after amoxicillinpseudolymphoma) .[1, 19].

The diagnosis of DRESS syndrome may be challenging. There is no diagnostic test for the disease and the diagnosis is mainly clinical. The Registry of Severe Cutaneous Adverse Reactions (RegiSCAR) scoring system [2] is used to support the diagnosis with DRESS syndrome and is based on clinical manifestations, organs affected, and the clinical course [20]. Our patient exhibited most of the clinical features of DRESS syndrome, including a fever ≥38.5°C, skin rash extending over more than 50% of the body surface, peripheral eosinophilia, and the liver injury 18 days following the initiation of an antibiotic treatment regimen. After excluding other potential causes, the RegiSCAR scoring system was used, and our patient had a total score of 5, fulfilling the criteria for the probable diagnosis of DRESS, as shown in Table 1.

Due to the rarity and clinical heterogeneity of DRESS syndrome, evidence-based management guidelines are lacking, and management is primarily based on experts' opinions [1, 12]. The prompt identification and withdrawal of the offending medication are the mainstay of therapy for all patients with DRESS syndrome. This action alone may be sufficient to resolve clinical and laboratory abnormalities, as was in our case. Further management is generally based on the severity of skin eruptions and other organ involvement. In mild disease (no organ involvement or only mild liver involvement), treatment is symptomatic. Systemic corticosteroids, immunosuppressive medications, and intravenous immunoglobulins are reserved for severe cases with significant visceral organ injury, particularly renal and/or pulmonary involvement [1, 11]. In our patient, progressive resolution of clinical features and laboratory abnormalities (inflammatory markers, eosinophils, and decrease in hepatic enzymes) was observed within two days of ceasing piperacillin-tazobactam and amikacin treatment, highlighting the importance of early recognition and prompt removal of inciting drugs to avoid harmful outcomes associated with DRESS syndrome.

In conclusion, the DRESS syndrome is a rare drug-induced hypersensitivity reaction that affects multiple organs and can be fatal if not recognized early. Due to its rarity in children and its symptoms overlapping with other commonly encountered pediatric conditions, pediatricians should have a high index of suspicion for children presenting with cutaneous and internal organ involvement and hematologic abnormalities after the initiation of an offending drug. Early recognition and prompt removal of the offending medication are critical for achieving the best outcomes.

## Figures and Tables

**Table 1:** Patient's diagnosis of DRESS syndrome based on the RegiSCAR system.

Clinical parameters	Present/absent (yes/no)	Patient score	RegiSCAR system
Fever (≥38.5)	Yes	0	No/unknown = −1
			Yes = 0

Lymphadenopathy	No	0	>1 cm, two sites; yes = 1
			No/unknow = 0

Eosinophilia	Yes	2	Eosinophil count of 0.7–1.49 × 10^9^ = 1
			Eosinophil count of ≥1.5 × 10^9^ = 2

Atypical lymphocyte	Unknown	0	Yes = 1
			No/unknown = 0

*Skin rash*			Suggestive features: ≥2 facial edemas, purpura, infiltration, desquamation (no = −1; unknown = 0; yes = 1)
(i) Extent ≥50% of BSA	Yes	1	
(ii) Rash suggestive of DRESS	Yes	1	

Organ involvement	Yes	1	1 point for each organ involvement, maximum score: 2

Disease duration ≥15 days	No	−1	No/unknown = −1
			Yes = 0

Skin biopsy suggesting DRESS	Not applicable	0	No: −1
			Yes/unknown: 0

Exclusion of other causes	Yes	1	1 point if ≥3 of the following tests were -ve: HAV, HBV, HCV, EBV, mycoplasma, chlamydia, ANA, blood culture

Total score		5	

Total score <2 = = no case. Total score 2–3 = possible case. Total score 4–5 = probable case. Total score >5 = definite case. BSA = body surface area; HAV = hepatitis A virus; HBV = hepatitis B virus; HCV = hepatitis C virus; EBV = Epstein-Barr virus; ANA = antinuclear antibodies.

**Table 2:** The trend in relevant laboratory findings in our patient during hospitalization and after discharge.

Laboratory test (normal values)	On admission	At diagnosis	Two days after antibiotic removal	At the fourth month follow-up visit
WBC (4–14 × 10^9^/L)	15.9	4.1	11	13.9
Eosinophils (0.1–0.7 cells × 10^9^/L)	0.4	1.8	1.2	0.5
CRP (<5 mg/L)	1.4	117	36	4.6
ALT (10–25 IU/L)	16	178	92	21
AST (23–46 IU/L)	35	686	90	40

WBC, white blood cell; CRP, C-reactive protein; ALT, alanine transaminase; AST, aspartate aminotransferase.

## Data Availability

The data presented in this study are available from the corresponding author upon request.

## Abbreviations

DRESS: Drug reaction with eosinophilia and systemic symptoms
CF: Cystic fibrosis
WBC: White blood cell
CRP: C-reactive protein
ALT: Alanine transaminase
AST: Aspartate aminotransferase
BUN: Blood urea nitrogen
BSA: Body surface area
HAV: Hepatitis A virus
HBV: Hepatitis B virus
HCV: Hepatitis C virus
EBV: Epstein–Barr virus
CMV: Cytomegalovirus
HHV: Human herpesvirus
ANA: Antinuclear antibodies.
